# Inhibition of Mitofusin-2 Promotes Cardiac Fibroblast Activation via the PERK/ATF4 Pathway and Reactive Oxygen Species

**DOI:** 10.1155/2019/3649808

**Published:** 2019-04-16

**Authors:** Yanguo Xin, Wenchao Wu, Jing Qu, Xiaojiao Wang, Song Lei, Lixing Yuan, Xiaojing Liu

**Affiliations:** ^1^Department of Cardiology, West China Hospital, Sichuan University, Chengdu 610041, China; ^2^Laboratory of Cardiovascular Diseases, Regenerative Medicine Research Center, West China Hospital, Sichuan University, Chengdu 610041, China; ^3^Department of Pathology, West China Hospital, Sichuan University, Chengdu 610041, China; ^4^Public Laboratory of West China Second University Hospital, Sichuan University, Chengdu 610041, China

## Abstract

Mitofusin-2 (Mfn2) is a key outer mitochondrial membrane protein, which maintains normal mitochondrial dynamics and function. However, its role in cardiac fibroblast activation remains poorly understood. In the present study, a rat model of transverse aortic constriction (TAC) was established to observe the cardiac fibroblast activation *in vivo*. TGF-*β*1 treatment for 24 hours was used to induce cardiac fibroblast activation *in vitro*. As a result, the expression of Mfn2 decreased in the hypertrophic heart tissues and cardiac fibroblasts treated with TGF-*β*1. siMfn2 and adenovirus were applied to mediate Mfn2 gene silencing and overexpression in cardiac fibroblasts to elucidate the relationship between Mfn2 and cardiac fibroblast activation, as well as the possible underlying mechanisms. Knockdown of Mfn2 further promoted TGF-*β*1-induced cardiac fibroblast activation, while forced expression of Mfn2 attenuated this pathological reaction. The PERK/ATF4 pathway, one of the branches of endoplasmic reticulum (ER) stress, was identified to be involved in this process. Knockdown and overexpression of Mfn2 lead to aggravation or alleviation of the PERK/ATF4 pathway. Blocking this pathway by silencing ATF4 with siATF4 attenuated the pathological process. During the activation of cardiac fibroblasts, knockdown of Mfn2 also increased the production of reactive oxygen species (ROS), while ROS scavenger N-acetyl-l-cysteine (NAC) could attenuate the effect caused by knockdown of Mfn2. Our data suggested that inhibition of Mfn2 could promote cardiac fibroblast activation by activating the PERK/ATF4 signaling pathway and increasing the generation of ROS.

## 1. Introduction

Cardiac hypertrophy occurring in pathological conditions (such as hypertension, valvular heart diseases, myocardial infarction, and cardiomyopathy) is an independent risk factor of cardiac morbidity and mortality. It is a complex cellular reprogramming process involving cardiomyocyte hypertrophy and cardiac fibroblast activation [1]. Cardiac fibroblasts represent about 2/3 of the cardiac cellular population and play a critical role in cardiac remodeling by regulating structural, biochemical, mechanical, and electrical properties of the heart [2, 3]. However, the mechanism of cardiac fibroblast activation is still largely unknown.

Evidence suggests that mitochondrial dynamics participates in the pathological process of cardiac hypertrophy [4, 5]. Mitochondrial dynamics is a collective term for mitochondrial movement, the count-balanced fusion-fission events, which regulate their morphology and functions. The fusion and fission events are regulated by specific GTP-dependent proteins that allow a tubule reticular networking morphology via fusion of the outer mitochondrial membrane (OMM) and inner mitochondrial membrane (IMM) fusion with redistribution of matrix components [6]. Interestingly, all of these mitochondrial dynamic-related proteins are present in the heart and vascular system but their roles there have only begun to be elucidated.

Mfn2, one of the mitochondrial fusion proteins located at the OMM of mitochondria, is a dynamic-like GTPase. Mfn2 plays a critical role in the maintenance of the mitochondrial network and normal function. Previous studies have demonstrated that overexpression of Mfn2 profoundly suppresses the mitogenic stimulus-evoked proliferation of vascular smooth muscle cells via inhibiting the Ras-ERK1/2 pathway [7]. Upregulation of Mfn2 inhibited angitensin-II induced myocardial hypertrophy by inhibiting Akt signaling [8]. Guan et al. reported that Mfn2 was a downstream mediator of miR-106a in pathological cardiac hypertrophy [9]. However, whether Mfn2 participates in the activation of cardiac fibroblasts has not been determined yet. Of particular note, Mfn2 is reported as the tethering of mitochondria to the endoplasmic reticulum (ER) to form the mitochondria-associated membranes (MAMs), which are unique structures crucial for mitochondrial bioenergetics. It is reported that the extent and length of MAMs depend on cellular conditions such as ER stress [10]. Therefore, Mfn2 may modulate ER stress beyond its function in regulating mitochondrial dynamics.

Our previous work has identified that ER stress and ROS are important mechanisms involved in cardiac hypertrophy [11–13]. ER stress is defined as the accumulation of misfolded proteins within the ER lumen, initiating three major pathways to restore ER homeostasis. These signaling pathways, known as unfolded protein response (UPR), comprise protein kinase RNA-like ER kinase (PERK)/activating transcriptional factor 4 (ATF4), inositol-requiring protein 1*α* (IRE1*α*)/Xbp1s, and activating transcriptional factor 6 (ATF6) pathways [14]. On the other hand, ROS is highly reactive in cardiomyocyte hypertrophy and even in the activation of cardiac fibroblasts via the NOX1/NADPH oxidase signaling pathway [15]. Based on the aforementioned findings, we postulated that upregulation of Mfn2 may protect cardiac fibroblast from activation, in part, via inhibiting ER stress and ROS.

Accordingly, this study was aimed at determining (1) the expression change of Mfn2 during the process of cardiac fibroblast activation, (2) the potential role of Mfn2 in the pathological process of cardiac fibrosis, and (3) whether ER stress and ROS are involved in the process of Mfn2-regulated cardiac fibroblast activation.

## 2. Materials and Methods

### 2.1. Animal Model of Transverse Aortic Constriction Surgery (TAC)

All animals used in our study received humane care, and this study was approved by the animal ethics committee of West China Hospital of Sichuan University (ethic number 2014003A). Transverse aortic constriction (TAC) in the rats is a widely used model for pressure overload-induced cardiac hypertrophy [16] and was used in our previous studies. Briefly, rats were anesthetized, intubated, and placed supine on a heated pat for mechanical ventilation. A suture was tied around a blunt 22-gauge needle placed on the aortic arch between the brachiocephalic and left carotid arteries, establishing a reproducible aortic stenosis. Control rats were sham operated. Four weeks after surgery, the operation's effects were evaluated by echocardiography before the rat hearts were dissected and snap frozen in liquid nitrogen and stored at -80°C until use.

### 2.2. Cell Culture and Pharmaceutical Treatments

The cardiac fibroblasts were obtained from decapitated 0- to 3-day-old Sprague-Dawley rats by collagenase II (0.05%) (Gibco) and trypsin (0.05%) digestion according to previous reports [17, 18]. Isolated cells from each digestion were pooled in DMEM (high glucose, BI, Israel) supplemented with 10% fetal bovine serum (FBS) (BI, Israel), penicillin (100 U/ml), and streptomycin (100 U/ml). The cell suspensions were centrifuged at 1200 rpm for 5 min, and the isolated cells were resuspended and seeded in a culture flask at 37°C and 5% CO_2_. After 90 min, for fibroblast adherence, cardiomyocytes were removed. Cardiac fibroblasts were passaged upon 90% confluency; after the first passage, cardiac fibroblasts were incubated with 1% BSA-DMEM for 4-6 h before being treated with the known cardiac fibroblast activation stimuli, TGF-*β*1 (10 ng/ml, PeproTech) for 24 h to induce cardiac fibroblast activation.

### 2.3. Small Interfering RNA (siRNA) Transfection

Cells were seeded on 6-well plates at 50-60% confluence before transfection. siRNAs for Mfn2 (siMfn2) were transfected into cardiac fibroblasts for 24 h using the transfection reagent riboFECT™ CP (RiboBio™, China) before stimulation with NAC (Selleck, S1623) for 2 h and then TGF-*β*1 for 24 h. Individual siRNAs (100 nM, RiboBio™, China), riboFECT™ CP buffer, riboFECT™ CP reagent, and DMEM were mixed, incubated at room temperature for 10-15 min, and then added to the cell cultures. All siRNA sequences are shown in Table 1.

### 2.4. Adenovirus Transduction

Recombinant Mfn2 adenoviruses designed and produced by Hanbio Biotechnology Co. Ltd. were constructed using the pHBAD-EF1-MCS-3flag-CMV-GFP vector. Adenoviruses containing rat Mfn2 gene, which encodes rat Mfn2 protein (AdMfn2) and sham adenovirus containing green fluorescent protein (AdGFP, Adctr) used in this study were propagated in HEK293 cells to a final titer of 1 × 10^10^. Cardiac fibroblasts were incubated with AdMfn2 and Adctr at a multiplicity of infection of 300. DMEM without FBS was used to dilute adenovirus. After incubation for 8 h, the medium was changed to serum-containing medium for 24 h followed by the treatments of stimuli.

### 2.5. Real-Time Polymerase Chain Reaction Analysis (qRT-PCR)

Gene expression was measured by quantitative qRT-PCR as our previous report [18]. Total RNA was extracted from cells with TRizol (Invitrogen, USA), and cDNA was synthesized using a reverse transcription (RT) kit (Toyobo, Japan). qRT-PCR was carried out on a CFX96 Real-Time PCR Detection System (Bio-Rad, USA) with fluorescence dye SYBR Green (SYBR Green Supermix kit, Bio-Rad, USA). Primer sequences were shown in Table 2. Normalization of gene expression was achieved by comparing the expression of *β*-actin for the corresponding samples. Relative fold expression values were determined applying the 2^−ΔΔCt^ threshold (Ct) method.

### 2.6. Intracellular ROS Measurement

Intracellular ROS production was determined by oxidative conversion of cell-permeable 20,70-dichlorofluorescein diacetate (H2DCFDA) (Sigma-Aldrich) to fluorescent dichlorofluorescein [11]. Cardiac fibroblasts were washed with serum-free DMEM and incubated with H2DCFDA (10 mM) for 60 min at 37°C in an incubator. Cells were then trypsinized (0.05% trypsin free of EDTA, Gibco) and resuspended in PBS, and the samples were analyzed by flow cytometry (FCM) (BD FACSCalibur) at excitation and emission wavelengths of 485 nm and 530 nm, respectively.

### 2.7. Immunofluorescence Staining and Confocal Imaging

Cardiac fibroblasts were plated in 24-well confocal plates; when reaching 60-70% confluent, cells were fixed with 4% paraformaldehyde for 30 min at 4°C and subsequently permeabilized with 0.5% Triton X-100 solution for 10 min at room temperature. Fixed cells were incubated with anti-*α*-SMA (1 : 200) or Mfn2 (1 : 200) primary antibody overnight at 4°C. The second antibody was Alexa Fluor 488 Goat Anti-Rabbit IgG (1 : 400, green fluorescence, Invitrogen, USA) or Alexa Fluor 594 Goat Anti-Rabbit IgG (1 : 400, red fluorescence, Invitrogen, USA). After incubation with the second antibody for 2 h at room temperature, rinse cells and incubate them with diluted DAPI for 10 min, away from light. Images were captured on a confocal inverted microscope for line scan analysis (FluoView FV1000, Japan).

### 2.8. EdU Proliferation Assay

Cells were seeded in 24-well plates to assay cell proliferation. Transfection or transduction and treatment of cells were performed as described above. Cell proliferation was detected using the incorporation of 5-ethynyl-2′-deoxyuridine (EdU) with an EdU Cell Proliferation Assay Kit (Ribobil*™*, China), according to the manufacturer's protocol. Cells were incubated with 50 *μ*M EdU for 1.5 h before fixation, permeabilization, EdU staining, and DAPI counterstaining. Images were collected using an Eclipse TE2000-U fluorescence microscope system (Nikon, Japan). For every group, 4 fields, or more than 800 cells, were evaluated in every experiment.

### 2.9. Protein Extraction and Western Blot Analysis

For tissue extraction, the frozen heart tissue was homogenized and lysed by a RIPA buffer (Beyotime) with protease and phosphatase inhibitor cocktail, subsequently sonicated for 15 s. For whole cell extraction, cells were lysed with RIPA buffer (Beyotime) with protease and phosphatase inhibitor cocktail. After centrifugation at 16000 × g for 10 min (4°C), the protein concentrations were subsequently determined using the BCA Protein Assay Kit (Thermo Fisher scientific). Then the protein samples (25 *μ*g) were subjected to SDS-PAGE, transferred to a PVDF membrane (Millipore, Bedford, MA), blocked with 5% skim milk in Tris-buffered saline and Tween 20 (TBST) solution for 2 h, and probed with corresponding primary antibodies at 4°C overnight, and HRP-conjugated secondary antibodies (1 : 3000) were incubated for 1 h. The antigen-antibody complexes were detected by enhanced chemiluminescence (ECL) (Bio-Rad). Images of the Western blot assay were carried out and analyzed using ChemiScope 6000 (CLINX, China). The protein levels were normalized to *β*-actin.

### 2.10. Wound Healing Assay

In order to verify the ability of migration, wound healing assay was performed on cardiac fibroblasts and cells were seeded in 6-well plates and grown to subconfluence. A scratch was then made in each well using a 200 *μ*l pipette tip, and the wounded monolayers were washed twice with PBS to remove cell debris and floating cells. The wounds (4 randomly selected points per wound) were photographed at the beginning (time 0 h) and then after 24 and 48 h under an inverted microscope with a digital camera, and the distance migrated by the cells was measured at the reference points by an image-processing software (Image-Pro Plus) [18].

### 2.11. Mitochondrial Morphology Observation by Transmission Electron Microscope (TEM)

Cells were seeded on 6-well plates at 50-60% confluence before siMfn2 transfection or adenovirus transduction. TEM was carried out according to a previous study [19]. In brief, after being fixed in cold 2.5% glutaraldehyde for 2 h at 4°C, cells were washed with PBS (0.2 mol/l, pH 7.4) for 2 h, fixed with 1% osmic acid for 2 h, and then washed six times with PBS for 10 min per wash. The samples were dehydrated with ethanol and cleaned with epoxypropane. They were embedded in EPON 812 overnight at room temperature. Ultrathin sections (40–60 nm) were cut (EM UC61rt, Leica) and stained with uranyl acetate/lead citrate. These sections were subsequently visualized using a transmission electron microscope (H-7650, Hitachi). We analyzed mitochondrial morphology according to previous publications; count measure and analysis on the TEM picture were carried out by ImageJ software, and we identified the length of mitochondria in the control group as the threshold value. At least three views of each group including more than 50 mitochondria (*n* > 50) were analyzed [20–22].

### 2.12. Statistical Analysis

All data are presented as mean ± SEM. Data were analyzed with SPSS22.0 software. Differences between groups were evaluated for significance using single-tailed Student's *t*-test of unpaired data or one-way analysis of variance (ANOVA). Bonferroni posttest analysis was used to compensate for multiple testing procedures. A value of *P* < 0.05 was considered significant.

## 3. Results

### 3.1. Mfn2 Expression Is Decreased in Heart Tissues of TAC Rats and Activated Cardiac Fibroblasts

**Figure 1:**
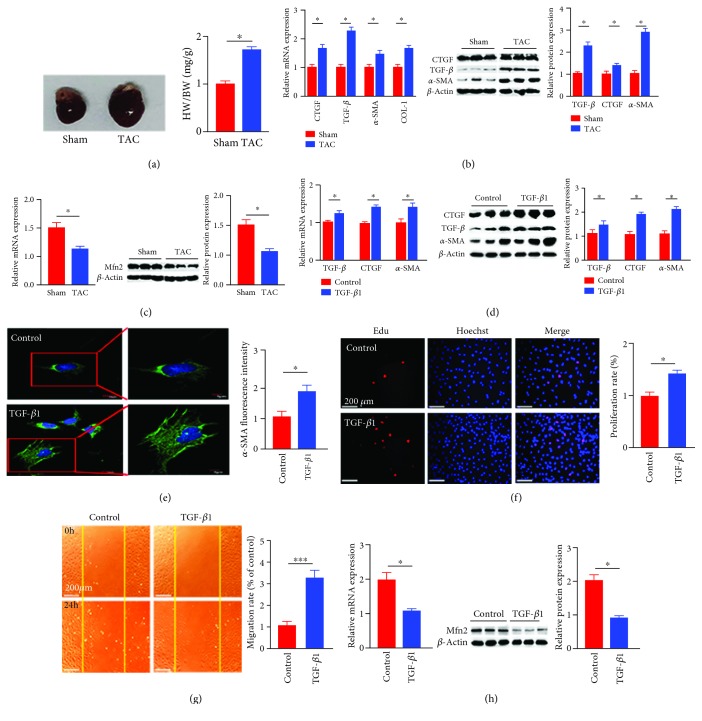
Mfn2 was downregulated in rat heart tissues with TAC surgery and in cardiac fibroblasts treated with TGF-*β*1. Rats were divided into two groups: sham and TAC. (a) Cardiac image and cardiac hypertrophy index, HW/BW (*n* = 6); (b) the mRNA and protein levels of CTGF, TGF-*β*, collagen1, and *α*-SMA in rat heart tissues (*n* = 6); (c) the mRNA and protein levels of Mfn2 in rat heart tissues (*n* = 6); cardiac fibroblasts were divided into two groups: control and TGF-*β*1 treatment: (d) the mRNA and protein levels of CTGF, TGF-*β*, and *α*-SMA in the process of cardiac fibroblast activation (*n* = 3); (e) immunofluorescence staining of *α*-SMA in cardiac fibroblasts (the red fluorescence indicated *α*-SMA and the blue fluorescence indicated the cell nucleus stained by DAPI, *n* = 60); (f) the proliferation rate of cells (the red fluorescence indicated EdU-incorporated cells and the blue fluorescence indicated the cell nucleus stained by DAPI, *n* = 120); (g) the migration activity of cardiac fibroblasts (determined by the number of protruding cells from the wound border); (h) the mRNA and protein levels of Mfn2 in the process of cardiac fibroblast activation (*n* = 3). Data in (a–h) are expressed as mean ± SEM. ∗ indicates *P* < 0.05, ∗∗ indicates *P* < 0.01, and ∗∗∗ indicates *P* < 0.001 vs. the sham or control groups.

Firstly, we evaluated the change of Mfn2 expression in TAC rats compared with that in sham-operated rats. Echocardiography indicators and HE staining suggested that cardiac hypertrophy is induced by the TAC model successfully in our previous papers [11, 18]. As shown in Figure 1(a), the left ventricular weight to body weight (HW/BW) (mg/g) ratio also implied that cardiac hypertrophy was induced in TAC models. Meanwhile, following TAC operation, the expressions of cardiac fibrosis markers, connective tissue growth factor (CTGF), collagen type I (COL-1), *α*-smooth muscle actin (*α*-SMA), and transforming growth factor *β* (TGF-*β*) mRNA, were increased by approximately 1.6-, 1.6-, 1.4-, and 2.2-fold, respectively, compared with those of the sham group. The protein levels of TGF-*β*, CTGF, and *α*-SMA also increased by about 2.1-,1.5-, and 2.8-fold, respectively (Figure 1(b)). Simultaneously, the mRNA and protein expressions of Mfn2 were downregulated approximately by 40% and 55%, respectively, in the TAC group compared with the sham group (Figure 1(c)).

Next, we detected Mfn2 expression in primary cultured cardiac fibroblasts treated with fibrotic agonist TGF-*β*1, 10 ng/ml for 24 h. As shown in Figure 1(d), compared with those of the TGF-*β*1 group, the mRNA levels of CTGF, *α*-SMA, and TGF-*β* were increased by 1.5-,1.4-, and 1.2-fold, respectively, in TGF-*β*1-stimulated cardiac fibroblasts; the protein expressions increased by 1.8-,1.5-, and 1.8-fold, respectively (Figure 1(d)). The confocal image analysis showed that the expression of *α*-SMA increased by 1.8-fold (Figure 1(e)), the proliferation of cardiac fibroblasts increased by 1.4-fold, and the migration rate increased by 3.2-fold (Figures 1(f) and 1(g)). In this condition, the mRNA and protein expressions of Mfn2 in fibrotic cardiac fibroblasts were decreased by almost 45% and 40%, respectively, compared with the untreated controls (Figure 1(h)). These results indicated that the expression of Mfn2 was decreased in the pathological process of cardiac fibroblast activation.

### 3.2. Mfn2 Plays a Protective Role in the Process of Cardiac Fibroblast Activation Induced by TGF-*β*1

**Figure 2:**
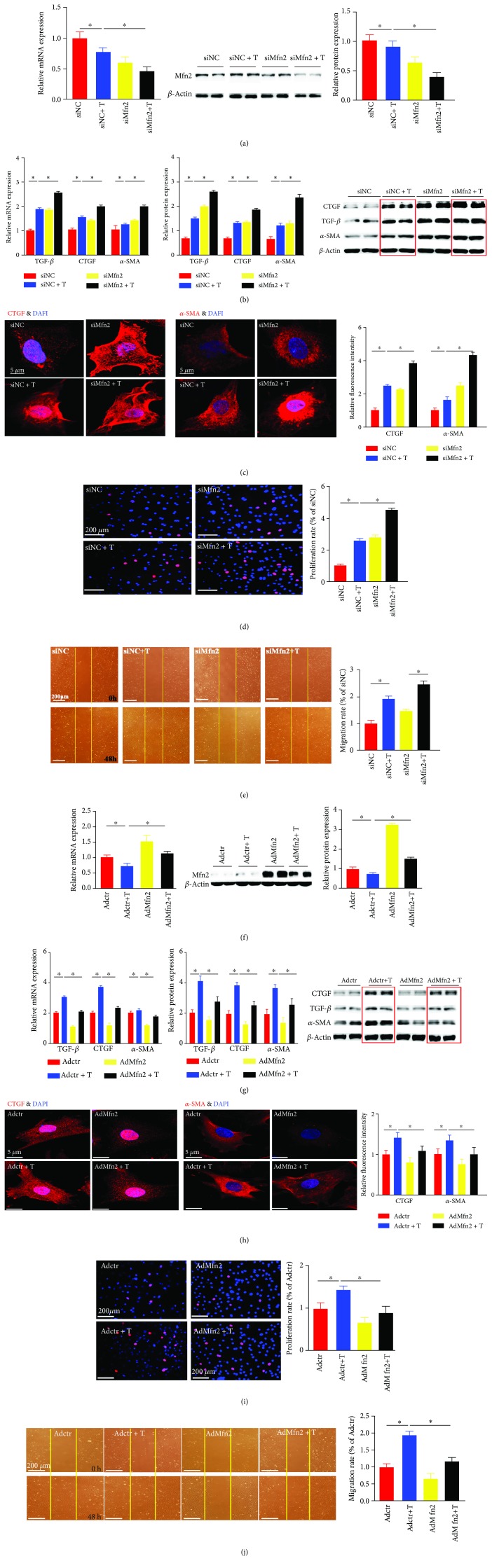
Mfn2 plays a protective role in the process of cardiac fibroblast activation induced by TGF-*β*1. (a) The mRNA and protein expressions of Mfn2 after siMfn2 transfection (*n* = 3); siMfn2 transfection in the presence or absence of TGF-*β*1; (b) the mRNA and protein levels of *α*-SMA, TGF-*β*, and CTGF in activated cardiac fibroblasts after transfection with siMfn2 (*n* = 3); (c) immunofluorescence staining of CTGF and *α*-SMA in cardiac fibroblasts (the red fluorescence indicated CTGF/*α*-SMA and the blue fluorescence indicated the cell nucleus stained by DAPI, *n* = 60) after transfection with siMfn2; (d) the proliferation rate of cardiac fibroblasts (the red fluorescence indicated EdU-incorporated cells and the blue fluorescence indicated the cell nucleus stained by DAPI, *n* = 120) after transfection with siMfn2; (e) the migration activity of cardiac fibroblasts (determined by the number of protruding cells from the wound border) after transfection with siMfn2; (f) the mRNA and protein expressions of Mfn2 after overexpression adenovirus transduction (*n* = 3); adenovirus transduction in the presence or absence of TGF-*β*1; (g) the mRNA and protein levels of *α*-SMA, TGF-*β*, and CTGF in activated cardiac fibroblasts after transduction with adenovirus (*n* = 3); (h) immunofluorescence staining of CTGF and *α*-SMA in cardiac fibroblasts (the red fluorescence indicated CTGF/*α*-SMA and the blue fluorescence indicated the cell nucleus stained by DAPI, *n* = 60) after transduction with adenovirus; (i) the proliferation rate of cardiac fibroblasts (the red fluorescence indicated EdU-incorporated cells and the blue fluorescence indicated the cell nucleus stained by DAPI, *n* = 120) after transduction with adenovirus; (j) the migration activity of cardiac fibroblasts (determined by the number of protruding cells from the wound border) after transduction with adenovirus. Data in (a–j) are expressed as mean ± SEM. ∗ indicates *P* < 0.05, ∗∗ indicates *P* < 0.01, and ∗∗∗ indicates *P* < 0.001 vs. the control groups.

To investigate whether Mfn2 played a potential role in cardiac fibroblast activation induced by TGF-*β*1, siMfn2, and Mfn2 overexpression adenoviruses were employed in our study. The results demonstrated that transfection with siMfn2 reduced the Mfn2 levels by about 40% at mRNA and protein levels (Figure 2(a)). Compared with the control group, downregulation of Mfn2 turned the cardiac fibroblasts into a more active phenotype since *α*-SMA, TGF-*β*, and CTGF were upregulated by 1.5-, 1.8-, and 1.4-fold, respectively, at mRNA level and 1.8-, 2.8-, and 1.9-fold at protein level (Figure 2(b)). Confocal images showed increased expressions of CTGF and *α*-SMA by 2.2-fold and 2.4-fold, respectively, compared with those in the control groups (Figure 2(c)). Silencing of Mfn2 could further promote an increase in the proliferation rate by about 2.5-fold and an increase in the migration rate of cardiac fibroblasts by 1.4-fold (Figures 2(d) and 2(e)). Our data demonstrated that downregulating the Mfn2 level further promoted the activation of cardiac fibroblasts.

To further investigate the role of Mfn2 in this pathological condition, overexpression of Mfn2 by adenovirus transduction was applied in our experiments. After transduction, Mfn2 was upregulated by almost 1.5-fold and 3.1-fold at the mRNA and protein levels, respectively, (Figure 2(f)), causing decreased mRNA expression of *α*-SMA (60%), CTGF (55%), and TGF-*β* (50%), compared with those of the control group (Figure 2(g)). Meanwhile the expressions of *α*-SMA, CTGF, and TGF-*β* were decreased by 25%, 30%, and 20%, respectively, at protein level (Figure 2(g)).

Confocal image also showed decreased expression of CTGF and *α*-SMA after Mfn2 adenovirus transfection by 20% and 25%, respectively (Figure 2(h)). Moreover, compared with the control group, the EdU-positive cells in the Mfn2 adenovirus group decreased by nearly 40%. Meanwhile, the migration ability of cardiac fibroblasts was reduced by 35% compared with the control group (Figures 2(i) and 2(j)).

All the above results demonstrated that Mfn2 might play a protective role in the process of cardiac fibroblast activation induced by TGF-*β*1.

### 3.3. Mitochondrial Morphology Is Impaired in Activated Cardiac Fibroblasts

**Figure 3:**
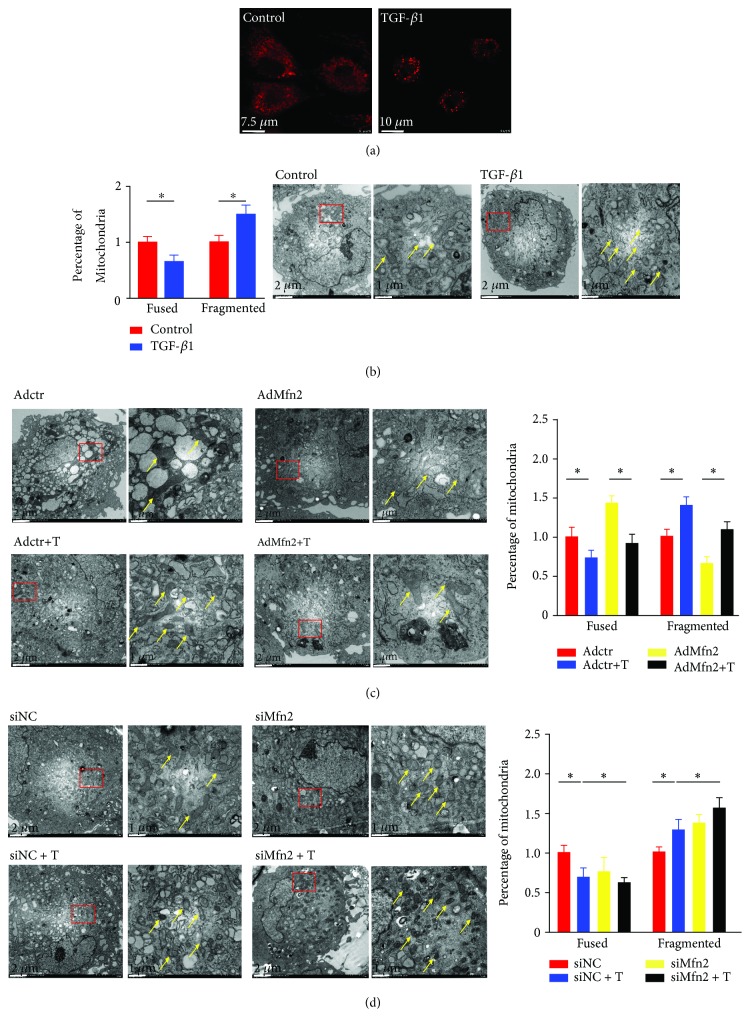
Mitochondrial morphology was impaired in activated cardiac fibroblasts. (a) MitoTracker analysis of cardiac fibroblasts treated with TGF-*β*1 for 24 h; (b) the morphology of fused and fragmented mitochondria in cardiac fibroblasts treated with TGF-*β*1 for 24 h; (c) the morphology of fused and fragmented mitochondria in cardiac fibroblasts with siMfn2 transfection in the presence or absence of TGF-*β*1; (d) the morphology of fused and fragmented mitochondria in cardiac fibroblasts with overexpression adenovirus transduction in the presence or absence of TGF-*β*1; data in (a–d) are expressed as mean ± SEM. The red arrow is for fused mitochondria; the yellow arrow is for fragmented mitochondria; ∗ indicates *P* < 0.05, ∗∗ indicates *P* < 0.01, and ∗∗∗ indicates *P* < 0.001 vs. the NC or NC+TGF-*β*1 groups.

Because Mfn2 is a protein located on the OMM of mitochondria, we observed the morphological changes of mitochondria in the process of cardiac fibroblast activation. In Figure 3(a), compared to those of the control group, MitoTracker imaging showed that fragmented mitochondria in cardiac fibroblasts increased after being treated with TGF-*β*1. The TEM results showed that after activation induced by TGF-*β*1, cardiac fibroblasts displayed fragmented mitochondrial morphology increasing by about 1.6-fold, while the number of fused mitochondria decreased about 30% (Figure 3(b)). After transduction with Mfn2 overexpression adenovirus, the percentage of fragmented mitochondria decreased by about 40% and that of fused mitochondria increased by 1.4-fold comparing to those of the control group (Figure 3(c)). Conversely, fused mitochondria decreased (40%) and fragmented ones increased (1.4-fold) after transduction with siMfn2 (Figure 3(d)).

### 3.4. Mfn2 Modulates ER Stress through Repression of the PERK/ATF4 Pathway in Activated Cardiac Fibroblasts

**Figure 4:**
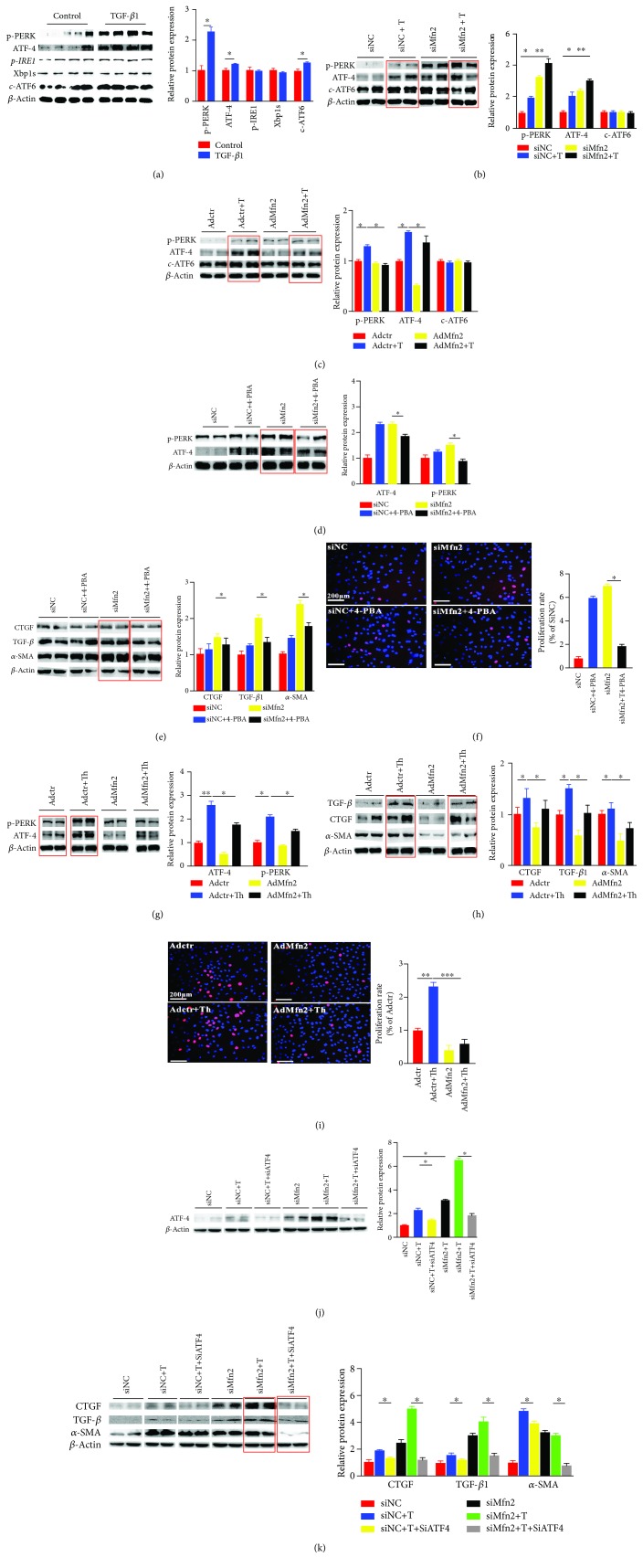
Mfn2 modulated ER stress through repression of the PERK/ATF4 pathway in activated cardiac fibroblasts. (a) Protein expressions of p-PERK, ATF4, p-IRE1*α*, Xbp1s, and c-ATF6 with TGF-*β*1 treatment in cardiac fibroblasts (*n* = 3); (b) protein expressions of p-PERK, ATF4, p-IRE1*α*, Xbp1s, and c-ATF6 in cardiac fibroblasts with siMfn2 transfection in the presence or absence of TGF-*β*1 (*n* = 3); (c) protein expressions of p-PERK, ATF4, p-IRE1*α*, Xbp1s, and c-ATF6 in cardiac fibroblasts with overexpression adenovirus transduction in the presence or absence of TGF-*β*1 (*n* = 3); (d) the protein level of the ER stress branch, the PERK/ATF4 pathway, after siMfn2 transfection (*n* = 3); siMfn2 transfection in the presence or absence of 4-PBA; (e) the protein levels of *α*-SMA, TGF-*β*, and CTGF in cardiac fibroblasts after transfection with siMfn2 (*n* = 3); siMfn2 transfection in the presence or absence of 4-PBA; (f) the proliferation rate of cardiac fibroblasts (the red fluorescence indicated EdU-incorporated cells and the blue fluorescence indicated the cell nucleus stained by DAPI, *n* = 120) after transfection with siMfn2; siMfn2 transfection in the presence or absence of 4-PBA; (g) the protein level of the ER stress branch, the PERK/ATF4 pathway, after overexpression adenovirus transduction (*n* = 3); adenovirus transduction in the presence or absence of thapsigargin; (h) the protein levels of *α*-SMA, TGF-*β*, and CTGF in cardiac fibroblasts after transduction with overexpression adenovirus (*n* = 3); overexpression adenovirus transduction in the presence or absence of thapsigargin; (i) the proliferation rate of cardiac fibroblasts (the red fluorescence indicated EdU-incorporated cells and the blue fluorescence indicated the cell nucleus stained by DAPI, *n* = 120) after transduction with adenovirus; overexpression adenovirus transduction in the presence or absence of thapsigargin; (j) protein expression of ATF4 with siMfn2 transfection followed with siATF4 transfection in the presence or absence of TGF-*β*1 in cardiac fibroblasts; (k) the protein levels of *α*-SMA, TGF-*β*, and CTGF in cardiac fibroblasts after siMfn2 transfection followed with siATF4 transfection in the presence or absence of TGF-*β*1 in cardiac fibroblasts; data in (a–k) are expressed as mean ± SEM. ∗ indicates *P* < 0.05, ∗∗ indicates *P* < 0.01, and ∗∗∗ indicates *P* < 0.001 vs. the NC or siMfn2 groups.

As we have identified the mitochondria morphology change with increased or decreased Mfn2 expression, we further explored the activation of ER stress pathways. After activation of cardiac fibroblasts induced by TGF-*β*1, PERK/ATF4, and c-ATF6 but not IRE1/Xbp1s, branches were upregulated (Figure 4(a)). Furthermore, the PERK/ATF4 pathway was further upregulated after silencing of Mfn2 with siMfn2 (Figure 4(b)); after overexpression of Mfn2, this signaling was further downregulated compared with the control group (Figure 4(c)).

To further identify that the PERK/ATF4 pathway was involved in the activation of cardiac fibroblasts, we employed ER stress inhibitor 4-PBA, ER stress activator thapsigargin, and specific ATF4 knockdown siRNA in our study. Our data indicated that after transfection with siMfn2 and followed with 4-PBA treatment, the expression level of PERK decreased by up to 70% compared with that of the siMfn2 group without 4-PBA (Figure 4(d)). Then we found that followed by blockade of the PERK/ATF4 pathway, the activation of cardiac fibroblasts was attenuated with the evidence that protein expressions of CTGF, TGF-*β*, and *α*-SMA decreased by about 20%, 30%, and 30%, respectively, comparing with those of the siMfn2 group (Figure 4(e)). Data about the proliferation rate according to EdU was consistent with the above results (Figure 4(f)). Conversely, after transduction with Mfn2 overexpression adenovirus, the protein expression levels of PERK and ATF4 in the AdMfn2 with thapsigargin (AdMfn2+Th) group increased by 1.5- and 3-fold, respectively, compared with those of the AdMfn2 group without thapsigargin (Figure 4(g)). With the activation of the PERK/ATF4 pathway, cardiac fibroblast activation was aggravated with the protein expressions of CTGF, TGF-*β*, and *α*-SMA increased by about 1.3-, 2.2-, and 0.8-fold, respectively, comparing with the siMfn2 group (Figure 4(h)). The activation and proliferation rates of cardiac fibroblasts also increased according to Western blot and EdU results (Figure 4(i)).

Furthermore, we applied specific ATF4 siRNA to confirm the above findings. The results demonstrated that compared with the siNC+TGF-*β*1 group, siATF4 reduced the ATF4 levels by 50% (Figure 4(j)). And specific silence of ATF4 receded the process of siMfn2-induced cardiac fibroblast activation with decreased protein expressions of CTGF, TGF-*β*, and *α*-SMA by 35%, 40%, and 45%, respectively (Figure 4(k)).

All the above data indicated that Mfn2 modulated ER stress through repression of the PERK/ATF4 pathway in the pathological process of cardiac fibroblast activation.

### 3.5. Mfn2 Regulates the Generation of ROS in Activated Cardiac Fibroblasts

**Figure 5:**
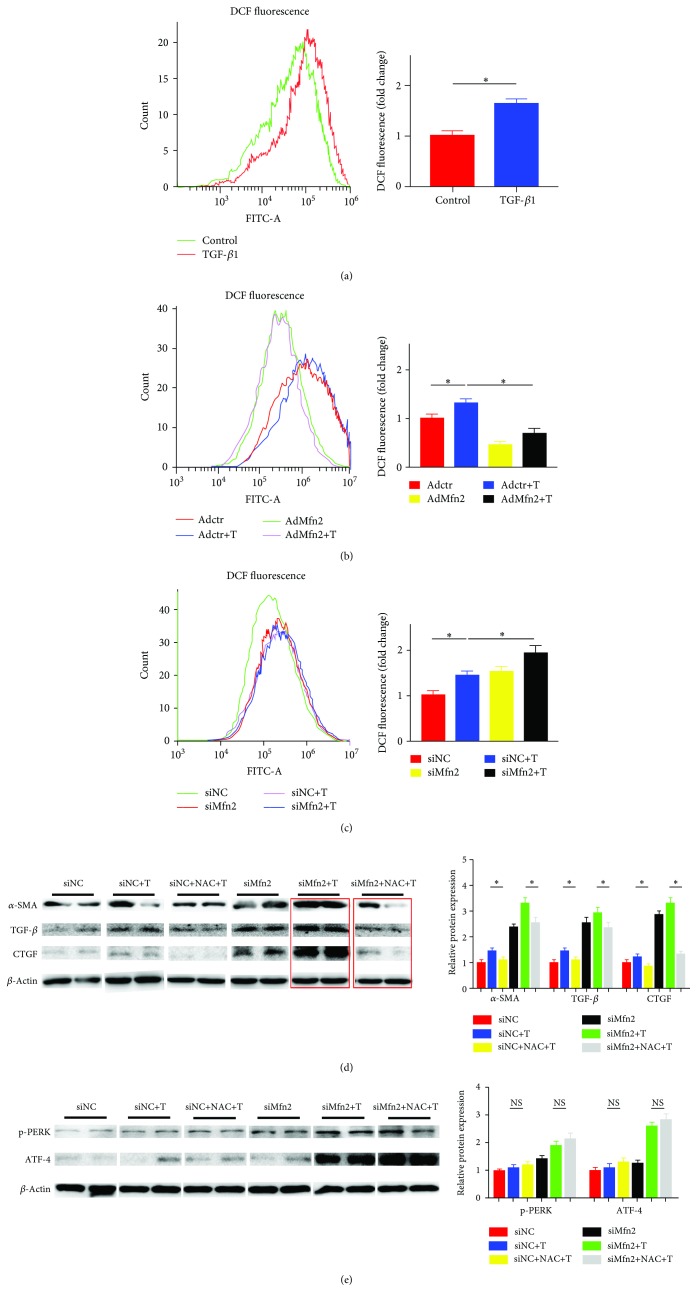
Mfn2 regulated the generation of ROS in activated cardiac fibroblasts. (a) The ROS level in cardiac fibroblasts treated with TGF-*β*1 for 24 h; (b) the ROS level in cardiac fibroblasts after overexpression adenovirus transduction; adenovirus transduction in the presence or absence of TGF-*β*1; (c) the ROS level in cardiac fibroblasts after siMfn2 transfection; siMfn2 transfection in the presence or absence of TGF-*β*1; (d) the protein levels of *α*-SMA, TGF-*β*, and CTGF in cardiac fibroblasts after transfection with siMfn2 (*n* = 3); siMfn2 transfection in the presence or absence of NAC. Data in (a–d) are expressed as mean ± SEM. NS: no significance. ∗ indicates *P* < 0.05, ∗∗ indicates *P* < 0.01, and ∗∗∗ indicates *P* < 0.001 vs. the NC groups.

To investigate the association between ROS and Mfn2 in cardiac fibroblast activation, we detected the ROS generation by flow cytometry. Compared with the control group, ROS generation was increased by about 1.6-fold in cardiac fibroblasts induced by TGF-*β*1 (Figure 5(a)). After that, we explored whether Mfn2 regulated cardiac fibroblast activation via ROS. According to Figures 5(b) and 5(c), ROS was decreased by 60% after overexpression of Mfn2 but increased by 1.4-fold after specific knockdown of Mfn2.

Further, we employed ROS scavenger, NAC, to figure out the association between Mfn2 and ROS. Cardiac fibroblasts were transfected with siMfn2 for 24 h before being treated with NAC and TGF-*β*1. Western blot was used to confirm the inhibition effect of siMfn2. The results demonstrated that after being treated with NAC, the activation of cardiac fibroblasts induced by siMfn2 and TGF-*β*1 was attenuated (Figure 5(d)). These results indicated that Mfn2 modulated the process of cardiac fibroblast activation by regulating the intracellular ROS level.

In order to figure out the potential link between ER stress and ROS in the process of cardiac fibroblasts, we detected the expression levels of the PERK/ATF-4 pathway with the treatment of NAC. Comparing with those of the control group, p-PERK and ATF-4 proteins showed no significant difference in NAC-treated cells (Figure 5(e)).

## 4. Discussion

**Figure 6:**
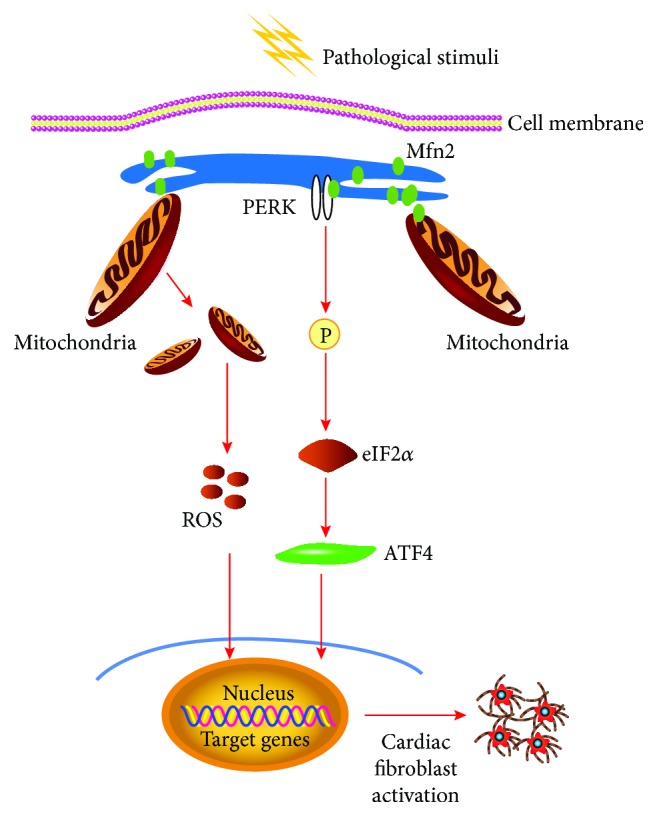
Schematic representation of the PERK/ATF4/ROS pathways through which Mfn2 inhibition triggers cardiac fibroblast activation. Upon pathological stimuli, gene silencing of Mfn2 reduced OMM Mfn2 expression, resulting in elevated expression levels of the PERK/ATF4 branch, and the generation of ROS ultimately leading to cardiac fibroblast activation.

In the present study, the role of Mfn2 in cardiac fibroblast activation was investigated. The major findings were illustrated in Figure 6.

Mfn2 is an important mitochondrial dynamin-related protein in maintaining the mitochondrial network and bioenergetics. Apart from its major involvement in mitochondrial fusion, dysfunction of Mfn2 is associated with various pathological conditions, including diabetes type 2 [23] and obesity [24]. Mfn2 is also known as hyperplasia suppressor gene (HSG). Previous studies have reported that Mfn2 has a potential role in regulating cell proliferation, apoptosis, and differentiation in many cell types [25–27]. Mfn2 expression is reduced in hypertrophic models [28]. In our study, the data of the TAC model showed that expression of Mfn2 decreased in hypertrophic rat hearts as early as four weeks after TAC surgery. Due to the fact that the lack of adeno-associated virus (AAV) transfected into cardiac fibroblasts *in vivo*, we isolated cardiac fibroblasts to verify the function of Mfn2 *in vitro*. Consistent with our *in vivo* results, our *in vitro* experiments demonstrated that Mfn2 expression decreased in cardiac fibroblasts treated with TGF-*β*1. Additionally, forced expression of Mfn2 could compensatory rescue cardiac fibroblast activation and the blockade of Mfn2 expression is harmful to cardiac fibroblasts.

Another major finding of the present study is that the PERK/ATF4 pathway contributes to the development of Mfn2-related cardiac fibroblast activation. PERK/ATF4 and ATF6 branches, but not IRE/Xbp1s, are activated along with the decreased expression of Mfn2. However, downregulation of Mfn2 by its specific siRNAs further promoted cardiac fibroblast activation via the PERK/ATF4, but not the ATF6, pathway. These findings are consistent with a published work indicating that Mfn2 modulated the UPR via repression of PERK [29]. But we did not observe the changes of the distance between ER and mitochondria and its association to the disturbance of ER stress; therefore, we would focus on this part in the future work. We also observed that with the stimulation of 4-PBA, the proliferation and transdifferentiation of cardiac fibroblast increased. In physiological conditions, blocking the ER stress pathways via its specific blocker 4-PBA would cause accumulation of unfolded or misfolded proteins [30], which may further cause the proliferation and transdifferentiation of cardiac fibroblasts.

Previous studies indicate that in response to ER stress, ROS are produced downstream of and as a consequence of the UPR, leading to cell death [31]. Conversely, ROS is a critical mediator of ER dysfunctions [32]. In our experiments, we found that treatment with NAC, the ROS scavenger, had no effect on the activation of the PERK/ATF4 pathway in the transdifferentiation of cardiac fibroblasts. We held the idea that there may be a more complicated relationship between ROS and other ER stress branches. Many drugs and natural products with modulating ROS generation can regulate ER stress [33, 34]. ROS and mitochondria have been identified to have interactions. Mitochondria were regarded as a source and target for ROS [35]. Although ROS-dependent modification of certain key signaling pathways is involved in the pathogenesis of cardiac fibroblast activation, the interaction between ROS and Mfn2 needs further study.

In conclusion, our data have demonstrated that Mfn2 plays a protective role in cardiac fibroblast activation and downregulation of Mfn2 promotes cardiac fibroblast activation through the PEAK/ATF4 and ROS pathways. These data provide insights into novel mechanisms with potential treatment strategies for cardiac hypertrophy.

## Figures and Tables

**Table 1:** siRNA sequences of Mfn2 and ATF4.

Names	Sequences
siMfn2	CCTCTCCTTTGGACTGTAT
siATF4	CCTCACTGGCGAGTGTAAA
Negative control (NC)	Supported by RiboBio™

**Table 2:** Primer sequences and amplicon sizes for real-time RT-PCR.

Genes	Primer sequence (5′-3′)
COL-1	F: 5′ACGTCCTGGTGAAGTTGGTC3′ R: 5′TCCAGCAATACCCTGAGGTC3′
CTGF	F: 5′CAGGGAGTAAGGGACACGA3′ R: 5′ACAGCAGTTAGGAACCCAGAT3′
*α*-SMA	F: 5′CCGAGATCTCACCGACTACC3′ R: 5′ATGCCACAGGATTCCATACCC3′
Mfn2	F: 5′CACTTGTCTTCCCCAATGAG3′ R: 5′GGAGAGGAAACCUGAC3′
*β*-Actin	F: 5′CCUCUCCUUUGGACUGUAU3′ R: 5′ATGCCACAGGATTCCATACCC3′
TGF-*β*1	F: 5′TGAGTGGCTGTCTTTTGACG3′ R: 5′ACTGAAGCGAAAGCCCTGTA3′
ATF4	F: 5′TGGAGUGACCGCUCACAUUU3′ R: 5′CCUCACUGGCGAGUGUAAA3′

## Data Availability

The accessibility data used to support the findings of this study were collected according with scientific research criteria and can be available from the corresponding author upon request.
