# Corrigendum to “Inhibition of Mitofusin-2 Promotes Cardiac Fibroblast Activation via the PERK/ATF4 Pathway and Reactive Oxygen Species”

**DOI:** 10.1155/omcl/9801041

**Published:** 2025-08-18

Y. Xin, W. Wu, J. Qu, et al., “Inhibition of Mitofusin-2 Promotes Cardiac Fibroblast Activation via the PERK/ATF4 Pathway and Reactive Oxygen Species”, *Oxidative Medicine and Cellular Longevity* 2019 (2019): 3649808, https://doi.org/10.1155/2019/3649808.

In the article, an image duplication was identified in Figure 1a with the Control and TGF-β1 of Xbp1s [1].

The authors explained that this was due to an error during the preparation of the manuscript and provided the correct image. The corrected Figure 1a is shown below:

The authors and the Editorial board agree to the publication of the corrigendum.

We apologize for this error.

## Figures and Tables

**Figure 1:**
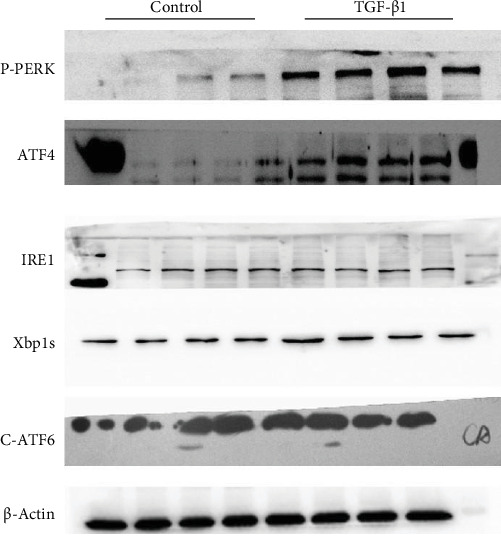
Mfn2 modulated ER stress through repression of the PERK/ATF4 pathway in activated cardiac fibroblasts. (a) Protein expressions of p-PERK, ATF4, p-IRE1α, Xbp1s, and c-ATF6 with TGF-β1 treatment in cardiac fibroblasts (*n* = 3); (b) protein expressions of p-PERK, ATF4, p-IRE1α, Xbp1s, and c-ATF6 in cardiac fibroblasts with siMfn2 transfection in the presence or absence of TGF-β1 (*n* = 3); (c) protein expressions of p-PERK, ATF4, p-IRE1α, Xbp1s, and c-ATF6 in cardiac fibroblasts with overexpression adenovirus transduction in the presence or absence of TGF-β1 (*n* = 3); (d) the protein level of the ER stress branch, the PERK/ATF4 pathway, after siMfn2 transfection (*n* = 3); siMfn2 transfection in the presence or absence of 4-PBA; (e) the protein levels of α-SMA, TGF-β, and CTGF in cardiac fibroblasts after transfection with siMfn2 (*n* = 3); siMfn2 transfection in the presence or absence of 4-PBA; (f) the proliferation rate of cardiac fibroblasts (the red fluorescence indicated EdU-incorporated cells and the blue fluorescence indicated the cell nucleus stained by DAPI, *n* = 120) after transfection with siMfn2; siMfn2 transfection in the presence or absence of 4-PBA; (g) the protein level of the ER stress branch, the PERK/ATF4 pathway, after overexpression adenovirus transduction (*n* = 3); adenovirus transduction in the presence or absence of thapsigargin; (h) the protein levels of α-SMA, TGF-β, and CTGF in cardiac fibroblasts after transduction with overexpression adenovirus (*n* = 3); overexpression adenovirus transduction in the presence or absence of thapsigargin; (i) the proliferation rate of cardiac fibroblasts (the red fluorescence indicated EdU-incorporated cells and the blue fluorescence indicated the cell nucleus stained by DAPI, *n* = 120) after transduction with adenovirus; overexpression adenovirus transduction in the presence or absence of thapsigargin; (j) protein expression of ATF4 with siMfn2 transfection followed with siATF4 transfection in the presence or absence of TGF-β1 in cardiac fibroblasts; (k) the protein levels of α-SMA, TGF-β, and CTGF in cardiac fibroblasts after siMfn2 transfection followed with siATF4 transfection in the presence or absence of TGF-β1 in cardiac fibroblasts; data in (a–k) are expressed as mean ± SEM. *⁣*_*∗*_ indicates *p* < 0.05,  _*∗*__*∗*_ indicates *p* < 0.01, and  _*∗*__*∗*_*⁣*_*∗*_ indicates *p* < 0.001 vs. the NC or siMfn2 groups.
